# HAPIscreen, a method for high-throughput aptamer identification

**DOI:** 10.1186/1477-3155-9-25

**Published:** 2011-06-03

**Authors:** Eric Dausse, Saïd Taouji, Laetitia Evadé, Carmelo Di Primo, Eric Chevet, Jean-Jacques Toulmé

**Affiliations:** 1 Inserm U869, Institut Européen de Chimie et Biologie, 2 rue Robert Escarpit, 33706 Pessac, France; 2 Inserm U1053 Avenir, Bat 1A, 146 rue Léo Saignat, 33076 Bordeaux, France; 3 Université de Bordeaux, 146 rue Léo Saignat, 33076 Bordeaux, France

## Abstract

**Background:**

Aptamers are oligonucleotides displaying specific binding properties for a predetermined target. They are selected from libraries of randomly synthesized candidates through an *in vitro *selection process termed SELEX (Systematic Evolution of Ligands by EXponential enrichment) alternating selection and amplification steps. SELEX is followed by cloning and sequencing of the enriched pool of oligonucleotides to enable comparison of the selected sequences. The most represented candidates are then synthesized and their binding properties are individually evaluated thus leading to the identification of aptamers. These post-selection steps are time consuming and introduce a bias to the expense of poorly amplified binders that might be of high affinity and are consequently underrepresented. A method that would circumvent these limitations would be highly valuable.

**Results:**

We describe a novel homogeneous solution-based method for screening large populations of oligonucleotide candidates generated from SELEX. This approach, based on the AlphaScreen^® ^technology, is carried out on the exclusive basis of the binding properties of the selected candidates without the needs of performing *a priori *sequencing. It therefore enables the functional identification of high affinity aptamers. We validated the HAPIscreen (High throughput APtamer Identification screen) methodology using aptamers targeted to RNA hairpins, previously identified in our laboratory. We then screened pools of candidates issued from SELEX rounds in a 384 well microplate format and identify new RNA aptamers to pre-microRNAs.

**Conclusions:**

HAPIscreen, an Alphascreen^®^-based methodology for the identification of aptamers is faster and less biased than current procedures based on sequence comparison of selected oligonucleotides and sampling binding properties of few individuals. Moreover this methodology allows for screening larger number of candidates. Used here for selecting anti-premiR aptamers, HAPIscreen can be adapted to any type of tagged target and is fully amenable to automation.

## Background

Aptamers are DNA, RNA or chemically-modified oligonucleotides selected from random pools of candidates containing up to 10^15 ^different sequences on the basis of their ability to bind to other molecules [1-3] or to catalyze predetermined reactions [4,5]. Within these molecules, intra-molecular folding generates up to 10^15 ^different structures that can be screened against a predetermined target for a chosen function, most often specific binding. Alternative steps of selection and amplification of selected candidates progressively enrich the pool in sequences that are exquisitely adapted to the recognition of the molecule of interest. To date aptamers have been selected against many different types of targets: small organic compounds, proteins, nucleic acids and complex scaffolds such as intact viruses or live cells [6,7]. Aptamer molecules share essential properties with antibodies such as high affinity and specificity. In addition, aptamers offer an alternative for the recognition of molecules such as toxins against which no antibody can be easily raised or for use under conditions that lead to protein denaturation [8,9]. At last, aptamers that are chemically synthesized on solid supports can readily be conjugated to different pending groups making them versatile tools for the labelling or the detection of their cognate target. They are of high interest for analytical technology [10-12] and numerous aptamer-based biosensors and probes have already been designed. These molecules are also of high potential value in medicine [13] as for instance an anti-VEGF aptamer has been recently approved by the Food and Drug Administration for the treatment of age-related macular degeneracy [14]. Several other molecules are currently being evaluated in clinical trials [15].

**Figure 1:**
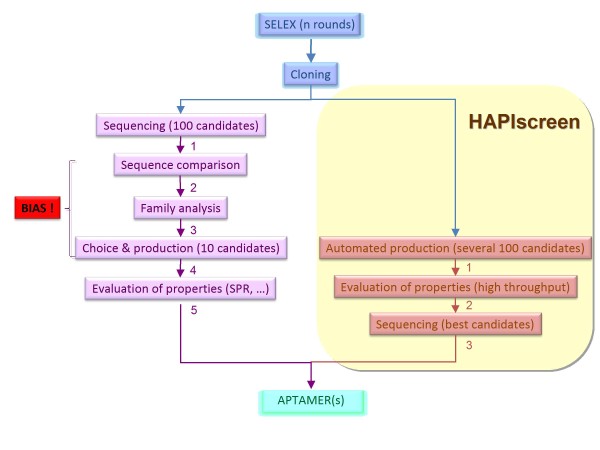
**Hapiscreen flowchart**. The different steps of the HAPIscreen methodology are indicated (right) in comparison to that of the standard SELEX method (left).

Aptamers are generally obtained by systematic evolution of ligands by exponential enrichment (SELEX) [16,17] even though a selection process without any amplification step (non-SELEX) has been previously described [18,19]. The current approaches require sequencing of the selected clones at the end of the in vitro selection. This is followed by a limiting step relying on sequence comparison and individual evaluation of few candidates for the identification of aptamers (Figure 1). This methodology is slow, incompatible with automation and is strongly biased since efficiently amplified poor affinity binders may mask low copy/high affinity aptamer candidates.

Given the increasing demand for aptamers [13] it would therefore be more efficient to screen directly for the desired property i.e. affinity of the target for a candidate (Figure 1). To this end we developed a functional screen downstream of the SELEX pipeline that relies on the AlphaScreen^® ^technology [20,21]. AlphaScreen^® ^is based on the use of both Donor (D, photosensitizer) and Acceptor (A, chemiluminescer) microbeads. Each bead is conjugated to one of the two potential interacting partners. A fluorescent signal is produced when both A and D beads are brought into proximity, thus reporting for the interaction between the biomolecular partners captured on the two beads [20]. This technology has been used for monitoring the interaction between various classes of molecules such as for instance protein/protein [22-24] or protein/RNA interactions [25]. In the present report, we demonstrate the interest of HAPIscreen (High throughput APtamer Identification screen), an AlphaScreen^®^-based method for the detection of aptamers targeted to different RNA hairpins of eukaryotic or viral origin.

## Results and Discussion

**Figure 2:**
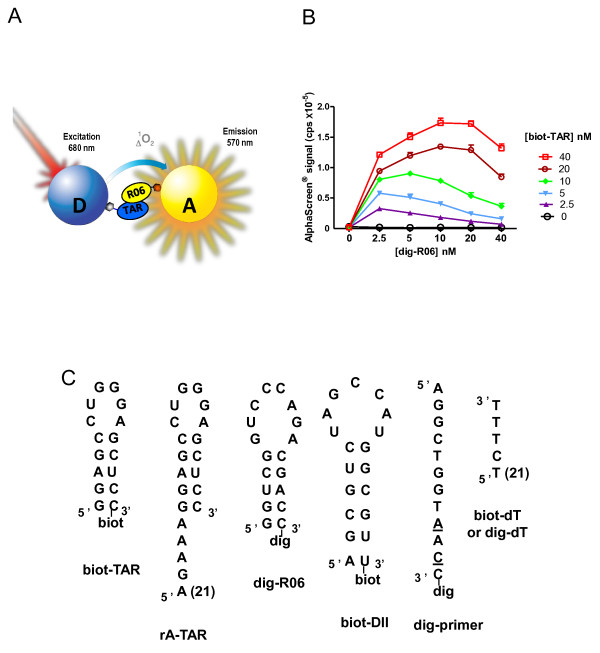
**HAPIscreen - proof of concept**. (**A**) Scheme of the assay setup using a digoxigenin-tagged aptamer (R06) and a biotinylated target RNA hairpin (TAR). The association of the two components is detected by using both Donor streptavidin (D) and Acceptor anti-digoxigenin (A) coated AlphaScreen^® ^beads. The production of singlet oxygen upon laser excitation by D-phtalocyanin is monitored by the fluorescence emission of A-rubrene beads. (**B**) Results obtained when increasing concentrations of dig-R06 were added to A and D beads for different biot-TAR concentrations (from 0 to 40 nM as indicated on the right). (**C**) Secondary structures and/or sequences of the top part of the *trans*-activating responsive (TAR) RNA element of HIV-1 (biot-TAR), 5' end-extended TAR (rA-TAR), RNA aptamer R06 (dig-R06), domain II of the HCV Internal Ribosome Entry Site (biot-DII), primer anchor (dig-primer). The latter was synthesized with 2'-O-methyl residues except at two positions (underlined) where Locked Nucleic Acid residues were introduced in order to promote hybridization and to increase complex stability. Oligod(T_3_CT_21_) anchor was used for capturing rA-TAR or the candidates from the M1 or M2 SELEX. The former were captured with biot-dT and the latter with dig-dT oligonucleotides, respectively.

In the AlphaScreen^® ^technology, Donor (D) and Acceptor (A) microbeads bear photosensitizing (phtalocyanin) and chemoluminescent molecules (rubrene), respectively. Laser excitation of the D beads at 680 nm causes ambient oxygen to be converted to the singlet state by phtalocyanin. Singlet oxygen species activate in turn a cascade of chemiluminescent reactions within the A beads leading to rubrene fluorescence detected between 520-620 nm using a photomultiplier tube-based microplate reader. A signal is produced when the A and D beads are brought into proximity (< 200 nm) through the interaction between two molecules of interest respectively immobilized on the two beads [20] (Figure 2A). Therefore monitoring the emission signal allows for the detection of complex formation. We used this technology for monitoring the interactions between a candidate aptamer (RNA oligomer) and its target (RNA hairpin). In the assay described here, D beads are conjugated to the target and A beads to the candidate to be tested. This is repeated for a number of candidates issued from a given SELEX round and the emission specific to each pair is measured. Candidates generating a signal are picked and those corresponding to the strongest signal subsequently sequenced for further characterization (Figure 1).

To demonstrate the interest of HAPIscreen for the detection of aptamers targeted to different eukaryotic RNA hairpins, we undertook a proof of concept phase showing its applicability to monitor a previously characterized HIV-1 RNA-aptamer association and to evaluate pools of HCV RNA-aptamer complexes. This was followed by the integration of a selection/evaluation process into an automated-SELEX/AlphaScreen^® ^pipeline allowing for identification of a large number of high affinity candidate aptamers to pre-microRNAs (premiRs).

### Monitoring RNA-RNA complexes with AlphaScreen^®^

In the proof of concept phase, we focused on a RNA-RNA complex that we previously characterized in great detail [26-28]. AlphaScreen^® ^was used to quantify the interaction between the *trans*-activating responsive (TAR) RNA element of HIV-1 [29] and a RNA aptamer, R06 identified from a random library of oligonucleotides (Figure 2). These two oligoribonucleotides adopt a stem-loop structure, display complementary sequences in the apical loop (Figure 2C) and were demonstrated to give rise to loop-loop (also called kissing) interactions. Increasing concentrations (0-40 nM) of digoxigenin-coupled R06 (dig-R06) were incubated in the presence of increasing concentrations of biotin-coupled TAR (biot-TAR), and constant amounts of streptavidin-D and anti-Dig-A beads. Such titration experiments were carried out at various biot-TAR concentrations ranging from 0 to 40 nM (Figure 2B). This revealed typical bell-shaped AlphaScreen^® ^signals showing a maximum for 40 nM biot-TAR and 10 nM dig-R06. Indeed as the anti-Dig-A-bead amount remains constant, addition of dig-R06 reaches a point at which it is no longer captured; consequently the excess of free dig-R06 competes with immobilized R06 for interacting with TAR captured on D-beads. This results in a decrease in fluorescence signal.

**Figure 3:**
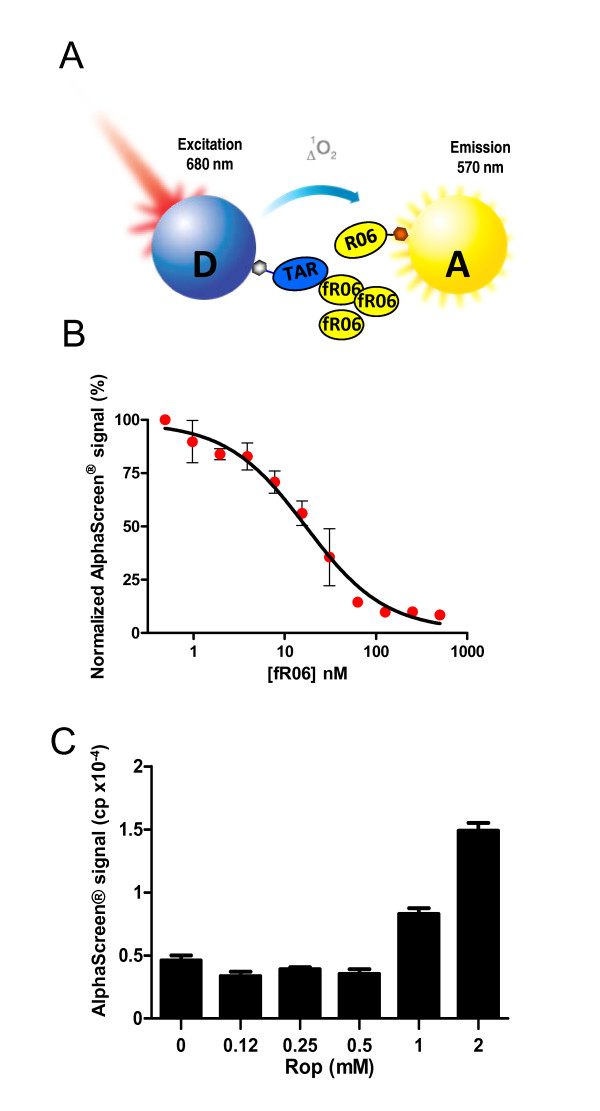
**AlphaScreen^®^-based characterization of TAR-R06 interaction**. (**A**) Competition assay setup. The assay was carried out as described in Figure 2A in the presence of untagged R06 (fR06). (**B**) Competition assay performed as described in **A**. Increasing concentrations of fR06 were added to the reaction containing 10 nM of dig-R06 and 40 nM of biot-TAR and the AlphaScreen^® ^signal was measured. Data are presented as percent of maximal signal (mean ± SD) and are representative of at least 3 independent experiments carried out in triplicate. (C) Rop binding assay to the TAR-R06 kissing complex. Alphascreen^® ^signal was obtained with a constant amount of biotinylated TAR and digoxiginated R06 (40 nM and 10 nM, respectively) as described in Materials and Methods, in the presence of increasing concentrations of the *E. coli *Rop protein. Data are representative of 3 independent experiments carried out in triplicate.

To demonstrate the specificity of the interaction, a competition assay was carried out in which increasing concentrations of unconjugated (free) R06 (fR06) were incubated in the presence of 10 nM dig-R06 and 40 nM biot-TAR (Figure 3A). As expected AlphaScreen^® ^signal decrease correlated with increasing concentrations of fR06 added to the reaction, leading to an apparent IC50 of 16.7 nM ± 1.7 (Figure 3B), a value reflecting an affinity in the same order of magnitude as that calculated using surface plasmon resonance (SPR) [26,28].

Using thermal denaturation which was monitored by UV absorption spectroscopy, SPR and gel-shift assays, we previously showed that magnesium ions stabilized the TAR-R06 complex [26]. In addition, we also used the *E. coli *protein ROP, which is involved in the control of the ColE1 plasmid copy number and specifically recognizes loop-loop complexes. We previously demonstrated that ROP was able to bind to the TAR-R06 complex [30,31]. In the AlphaScreen^® ^assay described above, increasing concentrations of ROP were added to biot-TAR-dig-R06 complexes in the absence or in the presence of 3 mM MgCl_2_. The addition of 1 mM ROP resulted in increased Alphascreen^® ^signal indicating the formation of a highly stable ROP-R06-TAR kissing complex (Figure 3C). A ~10 fold higher AlphaScreen^® ^signal was observed for this ternary complex in the presence of 3 mM MgCl_2 _compared to no magnesium (not shown). These experiments demonstrate that this methodology provides signals correlated to the affinity of the complex. Indeed, no signal was detected for R06 variants that do not complex with TAR, whereas conditions known to increase the interaction (addition of magnesium or ROP protein) resulted in increased fluorescence signals. Collectively these results demonstrate that AlphaScreen^® ^is suitable for monitoring the interaction between an aptamer and its target.

### Monitoring the evolution of SELEX RNA pools with HAPIscreen

**Figure 4:**
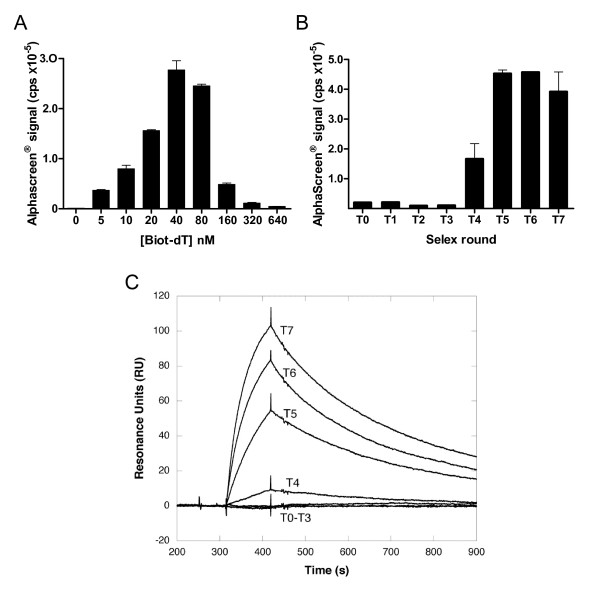
**Screening of oligonucleotide pools**. (**A**) TAR-R06 aptamer complexes were monitored as in Figure 2 except that a 5' extended TAR (rA-TAR) was immobilized on the beads through a biotinylated anchor oligonucleotide (biot-dT) (Figure 2C). Increasing concentrations of biot-dT are incubated in the presence of 40 nM rA-TAR and 10 nM dig-R06. The results are representative of three independent experiments carried out in triplicate (mean ± SD). Maximal AlphaScreen^® ^signal is obtained for stoichiometric concentrations of biot-dT and rA-TAR. (**B**) Monitoring the evolution of SELEX pools to the HCV IRES domain II (biot-DII) (Figure 2C). Aptamer populations from 7 SELEX rounds (T1 to T7) as well as the starting oligonucleotide pool (T0) were processed for AlphaScreen^® ^analysis as described in the Methods section. AlphaScreen^® ^signals are reported for each population as the mean of three independent experiments ± SD carried out in duplicate. (**C**) Monitoring the evolution of SELEX pools to the HCV IRES domain II (biot-DII) (Figure 2C) by SPR. Biot-DII was immobilized on a streptavidin-coated sensor chip (SAD200 m, XanTec Bioanalytics). The populations were prepared at 500 nM in the SELEX buffer and were injected over the surface at 20 μl/min.

Our objective was then to adapt this approach to the screening of large pools of SELEX-derived sequences. To this end, it was necessary to capture every selected candidate on the A beads. The above methodology in which biotinylated candidates are used would no longer be easily adapted to such a goal. We rather considered the use of a biotinylated anchor complementary to a pre-determined sequence on the candidates. Indeed every candidate contains fixed sequences flanking the random region, used in the SELEX process for the amplification of the selected candidates. An oligomer complementary to one of the flank efficiently allows for the capture of every candidate, at least those for which this region is not involved in a strong intra-molecular interaction. In order to validate this anchor-based approach a biotinylated oligod(T_3_CT_21_) (biot-dT) (Figure 2C) was assayed in the presence of 10 nM dig-R06 and 40 nM TAR bearing an oligor(A_21_GA_3_) tail (rA-TAR) (Figure 2C). Increasing concentrations of biot-dT (Figure 4A) led to a maximal AlphaScreen^® ^signal for rA-TAR concentrations ranging from 40 to 80 nM, in accordance with a stoichiometric association between TAR and the biot-dT anchor and the respective Donor and Acceptor bead capture capacities under the current assay conditions. Therefore the anchor-based methodology allows for monitoring aptamer-target interactions.

We then used such a strategy for evaluating the evolution of populations derived from 7 rounds of RNA SELEX previously carried out in our laboratory against the domain II (DII) of the Hepatitis C Virus mRNA internal ribosome entry site. This domain that folds as a hairpin with a 7 nt loop, was used as the target. *In vitro *selection against DII was previously demonstrated to produce high affinity aptamers [32]. To monitor the evolution of the RNA pool binding properties, candidates from the library (T0) and from rounds one to seven (T1-T7) were trapped on the acceptor beads using a digoxygenin-conjugated oligonucleotide, dig-primer (Figure 2C) complementary to their common 5' end. AlphaScreen^® ^signals obtained with D-beads carrying a 19 nucleotide long hairpin corresponding to the top part of DII were detected from round 4 and further increased at subsequent rounds, thus indicating the progressive enrichment of the population in strong binders (Figure 4B) in agreement with band shift assays [32] and SPR experiments carried out with bimolecular complexes formed between the immobilized HCV target and the SELEX pools (Figure 4C). These results demonstrate that the AlphaScreen^®^-based approach (HAPIscreen) could be undertaken for screening large collections of selected candidates using a unique anchor oligonucleotide.

### Identification of aptamers in RNA pools selected against premicroRNAs with HAPIscreen

**Figure 5:**
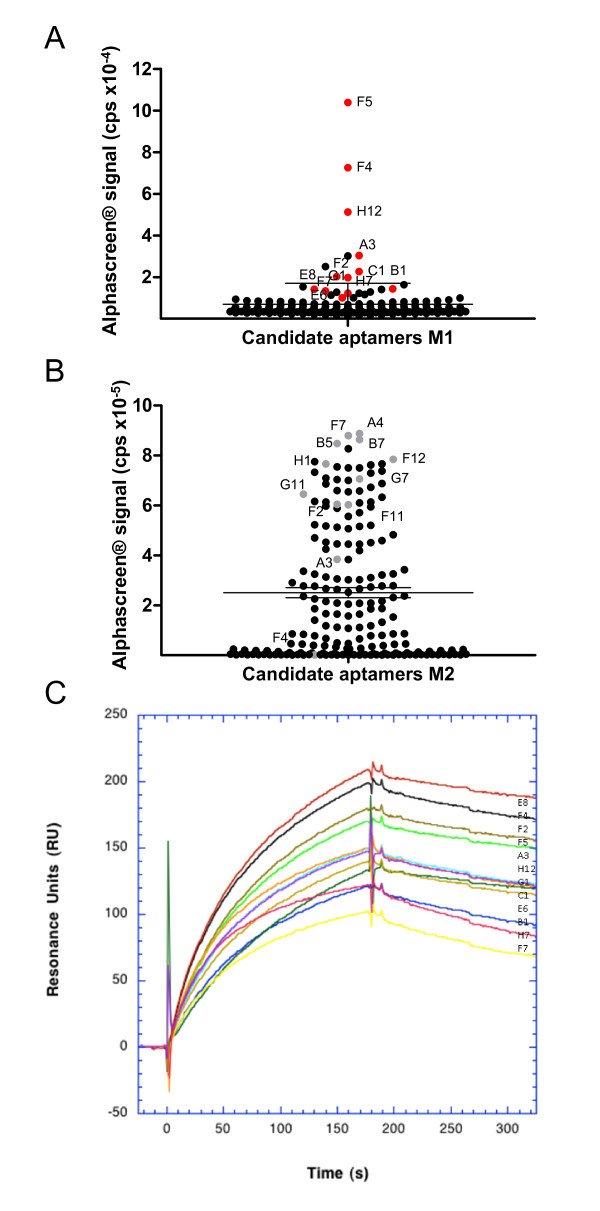
**Screening of oligonucleotide pools**. (**A, B**) AlphaScreen^®^-based analysis of individual candidates issued from SELEX M1 against a mixed target population a, b and c (**A**) or from SELEX M2 against the single target × (**B**). Error bars (horizontal bars in Figure 5 A, B) represent the mean ± SD values of three distinct AlphaScreen^® ^signal measurement for each SELEX population. For M2 the 75 percentile above and below the average is shown. (**C**) SPR analysis of twelve RNA candidates (corresponding to the red dots in Figure 5A) binding to a premiR target from SELEX M1. 400 RU of the biotinylated target were immobilised on a streptavidin-coated sensor chip (see Methods). The experiments were performed in the SELEX buffer at 23°C and the sensorgrams were collected at a flow rate of 20 μl/min. Candidates were injected at a concentration of 500 nM.

We then used HAPIscreen for evaluating the outcome of a SELEX experiment carried out using a RNA library against pre-microRNAs (premiRs). PremiRs display more or less perfect hairpin structures that are matured into functional miRs [33,34]. Aptamers raised against premiRs might consequently modulate miR interaction with proteins involved in the maturation process and impact on their regulatory function. Indeed oligonucleotides complementary to premiRs were shown to modulate the activity of miRs maturing enzymes [35,36]. SELEX was performed against two mixtures M1 and M2 of three human premiRs each (a, b, c and x, y, z, respectively) as described in Materials and Methods. At the end of seven SELEX rounds, the selected candidates were cloned and produced. Using HAPIscreen, candidates were trapped on the acceptor beads using digoxygenin-conjugated oligod(T_3_CT_21_) (dig-dT) (Figure 2C) complementary to their identical 3' end and individually assayed against each individual biotin-tagged target. One hundred ninety-two clones derived from the 7th round-enriched populations against either M1 or M2 were screened in duplicate blindly against the mixture of the three baits a, b and c or against the premiR × alone, respectively. It should be pointed out that the second experiment aims at identifying the partner actually targeted by a given aptamer selected against the mixture; repeating this experiment with immobilized y or z would allow the complete assignment of aptamers and targets. These experiments were carried out in 384-well plates and led to the identification of hits within both RNA populations (Figures 5A, B). Candidates from each selection were picked according to their high AlphaScreen^® ^signal (Figures 5A, B, red and grey dots, respectively) and tested individually by Surface Plasmon Resonance (Figure 5C). In the latter experiments, biotinylated pre-miRs a, b, c and x were individually immobilized on their respective sensor chip flow cells on which candidates were injected. Nine out of 12 (SELEX to × from M2) and 7 out of 12 (SELEX to a, b or c from M1) displayed evaluated K_D _values lower than or equal to 30 nM for unique-based or mixture-based target assays, respectively. There was a fair agreement between both SPR and the Alphascreen^® ^analyses even though the order of the signals was not rigorously the same with the two techniques (Figures 5A and 4C). We also tested the behaviour of 5 candidates that generated a low Alphascreen^® ^signal using SPR. This revealed that 4 out of the 5 candidates gave a weak or no resonance signal (not shown).

High affinity aptamers identified by Alphascreen^® ^were cloned and sequenced. As usual for *in vitro *selection we picked sequences displaying a high degree of similarity and identical motifs. Sequence differences likely account for the slight variation observed in Alphascreen^® ^or SPR signals. Finally, using MFold [37], aptamers from SELEX against M2 were predicted to adopt a consensus hairpin structure with a loop containing several nucleotides complementary to pre-miR loops. This suggested the formation of loop-loop aptamer/premiR complexes as previously described for TAR aptamers [26]. These aptamers and the aptamer-premiRs complexes are presently being characterized and will be described in a forthcoming manuscript. Using a similar approach, aptamers derived from SELEX against M1 also showed sequences complementary to premiR target loop, allowing for the formation of 8 potential adjacent base pairs but were not predicted to fold as hairpins.

## Conclusion

Herein, we have developed a method that overcomes the bias traditionally encountered in SELEX experiments. Usually candidates selected at the end of the process are cloned and sequenced. Sequences and/or predicted secondary structures are then compared in order to generate families and to choose few representatives that are then individually produced and characterized to identify aptamers (Figure 1). HAPIscreen bypasses the sequence comparison steps and directly allows for blind screening of aptamer candidates based on their exclusive binding properties rather than on sequence homologies. We demonstrate that HAPIscreen is a high throughput technology that can be used to analyze large collections of candidates. We used a 384-well plate format but HAPIscreen could as well be adapted to a 1536-well plate format. In addition, as the SELEX can be run simultaneously against a mixture of targets and the AlphaScreen^® ^analysis can be carried out against individual targets this predicts an increased discovery rate of aptamers. Moreover HAPIscreen is fully amenable to automation (currently the SELEX and the AlphaScreen^® ^steps are independently automated) and can be adapted for a wide range of targets due to the availability of different tags/beads. HAPIscreen also proved to be faster than traditional SELEX approaches as the process time was shortened by at least 50% from the isolated SELEX population to the identification of high affinity binders. The preparation of hundreds of candidates being achieved on an automated platform, this step is not time consuming. Finally, HAPIscreen potentially increases the chance of selecting orphan candidates (i.e. poorly amplified) by allowing the evaluation of larger aptamer collections. HAPIscreen therefore represents a major step forward in aptamer discovery and identification.

## Methods

### Oligonucleotide synthesis and purification

DNA primers and the biotinylated DNA anchor biot-dT, purchased either from Sigma or MWG Biotech, were purified by HPLC. All RNA targets and the digoxygenin 2'-O-methyl-LNA anchor (dig-primer) were chemically synthesized on an Expedite 8908 synthesizer (Applied Biosystems, USA) and purified by electrophoresis on denaturing 20% polyacrylamide, 7M urea gels. RNA candidates were synthesized by in vitro transcription using T7 RNA polymerase.

### AlphaScreen^® ^assays

The AlphaScreen^® ^technology was used to assess the interaction between candidate oligonucleotides derived from SELEX experiments, and biotinylated target. Binding assays were performed using white 384-well Optiplates (Perkin Elmer) in a total volume of 25 μl. The AlphaScreen^® ^reagents (anti-dig-coated Acceptor beads and streptavidin-coated Donor beads) were obtained from PerkinElmer. biot-TAR and dig-R06 (see figure 2C for oligonucleotide sequences) were prepared in a 10 mM sodium phosphate buffer, pH 7.2 at 20°C, containing 140 mM potassium chloride, 20 mM sodium chloride and 3 mM magnesium chloride. Prior to the experiments the RNA samples were heated in this buffer at 95°C for 1 min and 30 s and cooled down on ice for 10 min. The protein ROP, purified as previously described [30], was prepared in this buffer with or without magnesium chloride. For the analysis of SELEX populations, denaturation and refolding of the candidate aptamers and targets prior to reaction with the anchor (biot-dT) was performed in water at 65°C for 3 min or 80°C for 1 min, respectively. After denaturation, candidate aptamer and target were quickly cooled down to 4°C for 3 min and then equilibrated at room temperature (RT) for 5 min before adding the selection buffer (20 mM sodium acetate, 140 mM potassium acetate, 3 mM magnesium acetate, 20 mM HEPES; pH 7.4). Equal volumes (5 μl) of each partner, candidate and target, were incubated for 45 min at room temperature (RT), at final concentrations of 0.2 μM and 0.625 μM, respectively. In parallel Acceptor beads (20 μg/ml) were incubated with dig-primer (0.625 μM) for 1 h at room temperature in the selection buffer. Then, 10 μl of each of the interacting partners were added to the plate, after 45-min incubation at RT, 5 μl of Donor beads at a 20 μg/ml concentration were added to the mixture. All manipulations involving AlphaScreen^® ^beads were performed under subdued lighting. The plates were allowed to incubate either 1 h or overnight in the dark at room temperature. Light signal was detected by using an EnVision^® ^multilabel plate reader from PerkinElmer.

### *In vitro *selection

The RNA library used for the selections was obtained by transcription of the DNA library (5'-GTGTGACCGACCGTGGTGC-N30-GCAGTGAAGGCTGGTAACC-3') as previously described [38]. Two different primers P20 5'-GTGTGACCGACCGTGGTGC-3' and 3'SL containing the T7 transcription promoter (underlined) 5'-TAATACGACTCACTATAGGTTACCAGCCTTCACTGC-3' were used for PCR amplification. Oligonucleotide P20 was also used to prime reverse transcription. Selection steps were performed in the SELEX buffer (20 mM HEPES, pH 7.4 at 23°C, 20 mM sodium acetate, 140 mM potassium acetate and 3 mM magnesium acetate) at 23°C on an in-house assembled automated workstation (Tecan Freedom EVO 150). All steps (magnetic bead separation, vacuum purification, PCR amplification and transcription) were carried out in microplates. Two parallel SELEX, each against 3 target premiRs, constituting mixtures M1 and M2, were performed on the automated workstation. For each SELEX, 3 μM of the RNA library was heated at 80°C for 1 min, cooled at 4°C for 3 min, placed at room temperature for 5 min and mixed for the counter-selection with streptavidin-coated beads (50 μg of Streptavidin MagneSphere^® ^Paramagnetic Particles from Promega or 500 μg of Dynabeads M-280). RNA candidates not retained by the beads were then mixed and incubated for 10 min with 10 pmol of 3 different 3' end biotinylated premiRs that were previously immobilized on streptavidin beads. Unbound RNA was removed and the beads were washed twice with 100 μl of the SELEX buffer. The bound RNA candidates were eluted from the premiRs by heating for 1 min at 75°C in 50 μl of water. RNA candidates were reverse-transcribed with 200 units of M-MLV reverse transcriptase RNase H^- ^Point mutant (Promega) for 50 min at 50°C. The cDNA was amplified by PCR at 63°C with 20 units of the DNA polymerase AmpliTaq Gold™ (PerkinElmer) and the two primers P20 and 3'SL at 2 μM, during 25 cycles. RNA candidates were obtained by in vitro transcription of the PCR products with the Ampliscribe T7 transcription kit from Epicentre Biotechnologies. After 2 first manual and 5 automated rounds of selection against pre-miRs, carried out on an EVO150 (Tecan) in house-assembled robot, selected candidates were cloned using the TOPO TA cloning kit (Invitrogen).

### Synthesis, capture and sequencing of the candidates

In order to generate candidates for high throughput screening, we set up a second automated workstation (Tecan Freedom EVO 200) equipped with a thermal cycler, an orbital shaker, a magnetic particle separation module and a vacuum separation module. Three hundred and eighty four clones (192 clones from either M1 or M2 populations) from round 7 were produced blindly on this second workstation. Candidates were directly amplified from colonies with a 5' end oligod(T_21_CT_3_) (underlined) lengthened P20 5'-TTTTTTTTTTTTTTTTTTTTTCTTTGTGTGACCGACCGTGGTGC-3' and the 3'SL 5'-TAATACGACTCACTATAGGTTACCAGCCTTCACTGC-3' primers allowing the addition of an oligodA/T extension to the PCR products. RNAs produced by transcription of these PCR amplifications contained a 3' end oligorA tail that was used to capture them for the AlphaScreen^® ^tests with a digoxygenin-conjugated oligonucleotide (dig-dT) (Figure 2C). Candidates were sequenced using the BigDye Terminator v1.1 cycle sequencing kit (Applied Biosystems) according to the manufacturer's instructions.

### Surface Plasmon Resonance analyses

SPR experiments were performed at 23°C with a BIAcore™ 3000 apparatus. The biotinylated premiRs were immobilised at 50 nM (300 to 400 RU) on SA (BIAcore, GE Heathcare Life Sciences; Sweden) or SAD200 m sensor chips (XanTech Bioanalytics; Germany) coated with streptavidin. Aptamers were injected at 500 nM in the SELEX buffer at a 20 μl/min flow rate. After each injection of the candidates, the target-surface was regenerated with a 1 min pulse of a mixture containing 40% formamide, 30 mM EDTA and 3.6 M urea prepared in milli-Q water. The sensorgrams were analysed with the BIAeval software 4.1 as previously described [30]. Sensorgrams were double-referenced [39].

## Competing interests

The authors declare that they have no competing interests.

## Authors' contributions

ED carried out, designed and analyzed the SELEX experiments. ST designed and performed the Alphascreen^® ^experiments and analyzed the data. LE performed the manual and automated selection. CDP designed the interaction models and participated in the SPR measurements. EC supervised the Alphascreen^® ^measurements. JJT conceived the study and participated in its coordination. EC and JJT drafted the manuscript. All authors read and approved the final manuscript.
